# Small-scale gene duplications played a major role in the recent evolution of wheat chromosome 3B

**DOI:** 10.1186/s13059-015-0754-6

**Published:** 2015-09-09

**Authors:** Natasha M. Glover, Josquin Daron, Lise Pingault, Klaas Vandepoele, Etienne Paux, Catherine Feuillet, Frédéric Choulet

**Affiliations:** INRA UMR1095 Genetics, Diversity and Ecophysiology of Cereals, 5 chemin de Beaulieu, 63039 Clermont-Ferrand, France; University Blaise Pascal UMR1095 Genetics, Diversity and Ecophysiology of Cereals, 5 chemin de Beaulieu, 63039 Clermont-Ferrand, France; Present address: Bayer CropScience NV, Technologiepark 38, 9052 Ghent, Belgium; Department of Plant Systems Biology (VIB) and Department of Plant Biotechnology and Bioinformatics (Ghent University), Technologiepark 927, 9052 Ghent, Belgium; Present address: Bayer CropScience, 3500 Paramount Parkway, Morrisville, NC 27560 USA

## Abstract

**Background:**

Bread wheat is not only an important crop, but its large (17 Gb), highly repetitive, and hexaploid genome makes it a good model to study the organization and evolution of complex genomes. Recently, we produced a high quality reference sequence of wheat chromosome 3B (774 Mb), which provides an excellent opportunity to study the evolutionary dynamics of a large and polyploid genome, specifically the impact of single gene duplications.

**Results:**

We find that 27 % of the 3B predicted genes are non-syntenic with the orthologous chromosomes of *Brachypodium distachyon*, *Oryza sativa*, and *Sorghum bicolor*, whereas, by applying the same criteria, non-syntenic genes represent on average only 10 % of the predicted genes in these three model grasses. These non-syntenic genes on 3B have high sequence similarity to at least one other gene in the wheat genome, indicating that hexaploid wheat has undergone massive small-scale interchromosomal gene duplications compared to other grasses. Insertions of non-syntenic genes occurred at a similar rate along the chromosome, but these genes tend to be retained at a higher frequency in the distal, recombinogenic regions. The ratio of non-synonymous to synonymous substitution rates showed a more relaxed selection pressure for non-syntenic genes compared to syntenic genes, and gene ontology analysis indicated that non-syntenic genes may be enriched in functions involved in disease resistance.

**Conclusion:**

Our results highlight the major impact of single gene duplications on the wheat gene complement and confirm the accelerated evolution of the *Triticeae* lineage among grasses.

**Electronic supplementary material:**

The online version of this article (doi:10.1186/s13059-015-0754-6) contains supplementary material, which is available to authorized users.

## Background

Gene duplication is a major source of species adaptation, providing raw genetic material for functional diversification. The duplication of genes, along with alternative splicing, exon shuffling, and epigenetic regulation, has been shown to contribute to the vast complexity observed among eukaryotic genome architectures [1–4]. There are several types of gene duplication: large-scale, such as whole-genome duplication, and small-scale, where only one or a few genes are duplicated. Numerous marker-based comparative studies have demonstrated that grass genomes have a high degree of conserved synteny (homologous genes located on syntenic blocks between species) and collinearity (conserved gene order within syntenic blocks) [5–8]. Furthermore, access to the sequences of the rice, sorghum, maize, and *Brachypodium* genomes has enabled comparative analyses at a higher resolution [9–12], revealing that although synteny is well-conserved between orthologous grass chromosomes, many micro-rearrangements (including single gene duplications, insertions, and deletions) have disrupted the collinearity.

Hexaploid bread wheat (*Triticum aestivum* L.; 2n = 6x = 42; AABBDD) originated from two recent hybridizations between three diploid progenitors, donors of the A, B, and D subgenomes, which diverged an estimated 6.5 MYA [13]. The first hybridization occurred <0.8 MYA between the diploid donors of the A and B genomes, whose closest extant representatives are *Triticum urartu* (A genome) and *Aegilops speltoides* (S genome related to the B genome). It formed the allotetraploid *Triticum turgidum* that hybridized <0.4 MYA with the ancestor of *Aegilops tauschii* (D genome). Given its hexaploid composition, size of 17 Gb, and a percentage of transposable elements close to 90 % [14], the bread wheat genome is an interesting model to study the evolution of complex genomes and the impact of allopolyploidy on genome structure evolution and the fate of duplicated genes.

Several previous studies have estimated the proportion of non-syntenic genes in the wheat genome with model grass species to range from one-third to two-thirds of the genes. However, without access to a complete genome sequence, these analyses were based on ESTs mapped to genetic bins [15–17] or a subset of genomic sequence data from the wheat physical map [18]. In the closely related barley (*Hordeum vulgare*; wheat/barley divergence estimated at approximately 11.6 MYA [19]), a higher degree of synteny with wheat has been described, with less than one-third of the genes estimated as non-syntenic [18, 20, 21]. Additionally, comparative analyses using the physical map of the D genome progenitor, *Ae. tauschii*, led to the estimation that 26 % of the genes are non-collinear with *Brachypodium* [22]. Finally, in a study based on sequencing 2 % of the wheat 3B chromosome, Choulet *et al.* showed that 48 % of the genes are non-collinear with rice, *Brachypodium*, and sorghum [23]. Thus, there is convergent evidence to suggest that the *Triticeae* lineage, and wheat in particular, underwent accelerated evolution via gene duplication and movement. This is further evidenced by the higher number of inversions and translocations observed in *Ae. tauschii* compared to *Brachypodium*, sorghum and rice [22, 24], and by the elevated rate of alternative splicing and codon substitution observed in wheat [25].

Recently, we produced a high quality reference sequence of the wheat 3B chromosome [14]. This reference sequence was assembled into a pseudomolecule of 774 Mb, with 7,264 predicted protein-coding genes. In this study, we used the chromosome 3B reference sequence and the chromosome survey sequence assemblies of the bread wheat genome [26] to conduct a deeper analysis on the origin and fate of non-syntenic genes at the whole genome level as well as to study their distribution along a chromosome. Our results indicate that wheat has a higher duplication/fixation rate of non-syntenic genes compared to other grass species, and a selection for non-syntenic genes insertions in distal regions of the chromosomes. Compared to syntenic genes, non-syntenic genes have a more specific expression pattern, more relaxed selection pressure, and are enriched in functions that may provide adaptive advantages.

## Results

### Conserved genes in wheat and related model grasses

**Fig. 1:**
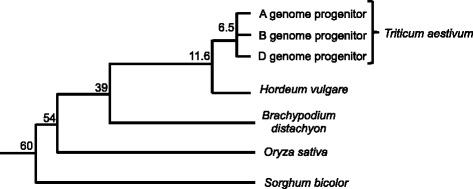
Phylogeny of the model grass species used in this study. Dating information (in MYA) was taken from [12, 13, 19]

In this study, we compared the wheat 3B chromosome reference sequence with the genomes of three related species: *Brachypodium distachyon*, *Oryza sativa* (rice), and *Sorghum bicolor* (sorghum), representing the *Pooideae*, *Ehrhartoideae*, and *Panicoideae* clades, respectively (Fig. 1). These species were chosen to explore the evolutionary dynamics of the highly complex and polyploid wheat genome compared to smaller, more compact model grass genomes. We verified the syntenic relationships between wheat chromosome 3 (Ta3B), rice chromosome 1 (Os1), sorghum chromosome 3 (Sb3), and the distal regions of *Brachypodium* chromosome 2 (Bd2) [12, 15, 27–29] and delineated their exact borders using EnsemblPlants Synteny viewer [30] (Additional file 1: Figures S1-S4).

**Fig. 2:**
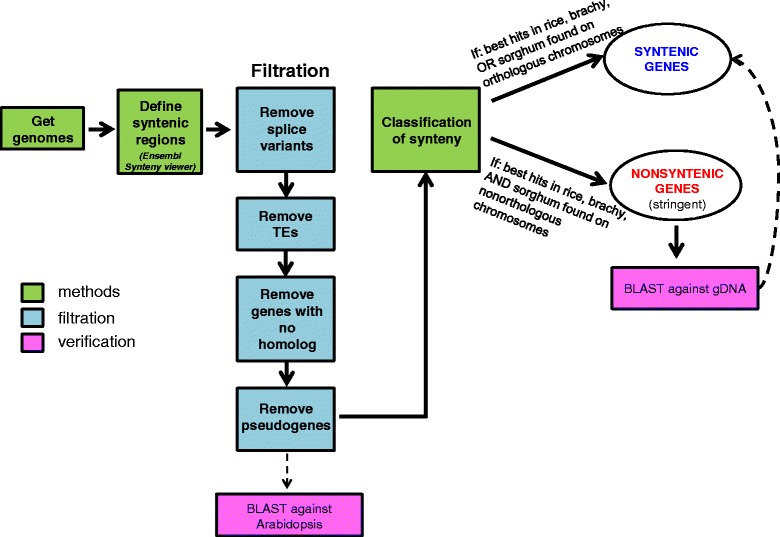
Methodology applied for classifying syntenic and non-syntenic genes

Since different methods of genome annotation can result in spurious gene predictions [31], we applied a filtration process to define a gene set that could be compared between species (for a flow chart of the methodology, see Fig. 2). We first discarded alternative splice variants in each genome, taking the longest as the representative. Second, we removed transposable element (TE)-related genes from our dataset. For rice, *Brachypodium*, and sorghum, this was done based on the annotation summary files downloaded for each genome (genes either already classified as TEs, or having ‘transposon’ in their description). For wheat 3B, we used the TE annotation performed by Daron *et al.* [32]. Third, we removed potentially mispredicted genes by only including genes for which we could find homology in at least one of the other species used in the study. Finally, in order to focus on functional genes, we removed predictions annotated as pseudogenes.

This filtration process allowed us to work on a ‘core’ gene set while removing mispredictions and/or potential lineage-specific genes. The core gene set consisted of 5,125, 3,804, 3,582, and 4,023 genes on the orthologous chromosomes of wheat 3B, *Brachypodium 2*, rice 1, and sorghum 3, respectively (Table 1). These results indicate that *Brachypodium*, rice, and sorghum have a similar number of genes in their core gene set (mean 3,803; Table 1) whereas wheat chromosome 3B carries 35 % more genes (5,125) than would be expected by comparison with the other grasses used in this study. We are confident that these additional approximately 1,000 genes in wheat are likely functional protein-coding genes because of the rigorous annotation process that was used for chromosome 3B based on [14, 33]. This includes: (1) training *ab initio* predictors based on thousands of wheat genes in order to improve the accuracy; (2) combining evidence from different methods of prediction and selecting the best gene model at a given locus based on a scoring system; (3) validation of 59 % of gene predicted splice sites based on transcript evidence (RNAseq, ESTs, mRNA); and (4) manual curation of 48 % of the 3B gene predictions. Moreover, 95 % of the 5,125 wheat core genes have significant sequence similarity to genes in the well-curated *Arabidopsis thaliana* genome, indicating these are likely to be real genes rather than mispredictions (Additional file 1: Table S1).

**Table 1:** Filtration results for the four species compared in this study

Species	Chr.	Number of genes (no ASVs or TEs)	Number of genes with at least 1 homolog	Number of genes after removing pseudogenes
*Triticum aestivum*	3B	7,703	6,254	5,125
*Brachypodium distachyon*	2	4,293	3,804	3,804
*Oryza sativa*	1	5,070	3,882	3,582
*Sorghum bicolor*	3	4,555	4,023	4,023

Sequential process of filtration, starting with the total numbers of genes in the syntenic regions of each chromosome after removing transposon related genes and alternative splice variants (ASVs). The numbers of genes with at least 1 homolog are those with a significant BLAST hit (>35 % amino acid identity and >35 % gene overlap) in at least one of the other species

These results demonstrate that wheat chromosome 3B has an increased number of genes in the core gene set compared to other species, and thus provide a first indication for an accelerated evolution via gene duplication of the gene repertoire in the wheat lineage.

### High rate of inter-chromosomally duplicated (non-syntenic) genes in wheat

We examined synteny along chromosome 3B and the orthologous regions in the other grasses. Orthologous gene pairs found in syntenic counterparts between at least two species were classified as syntenic genes (Fig. 2). For example, for each of the filtered genes of wheat, if the best BLAST hits in rice, *Brachypodium*, or sorghum were found on orthologous chromosomes, the wheat gene was considered syntenic. In contrast, non-syntenic genes were defined as genes having their best BLAST hits located on non-syntenic chromosomes in all the species compared (that is, if the best blast hits in rice, *Brachypodium*, and sorghum were found on non-orthologous chromosomes, the wheat gene was considered non-syntenic) [14].

Thus, non-syntenic genes originate from duplication and/or translocation having occurred in a lineage-specific manner. Finally, in order to be certain that we were not overestimating the number of non-syntenic genes simply due to a lack of a predicted ortholog in the gene annotation, we searched for sequence similarity of the non-syntenic genes of each species on the genomic sequence (pseudomolecules) of orthologous chromosomes in the other species. This verification step allowed us to reclassify on average 2.25 % of the core genes of each species from non-syntenic to syntenic, and to confirm that the remaining non-syntenic genes are indeed not present in orthologous locations rather than simply not predicted (Additional file 1: Table S2).

While approximately 10 % of the conserved genes in Bd2, Os1, and Sb3 were found to be non-syntenic, 27 % (1,397 genes) of the filtered Ta3B genes were classified as non-syntenic (Table 2). Based on recent divergence time estimates between the three last common ancestors of the species studied here, non-syntenic genes have been inserted in the past 39, 54, and 60 MY for wheat/*Brachypodium*, rice, and sorghum, respectively (Fig. 1) [12]. Taking these divergence times into consideration, we calculated the rates of non-syntenic genes insertion/fixation and found that for Os1, Bd2, and Sb3, they were highly similar, ranging from 1.7 × 10^−3^ to 2.3 × 10^−3^ locus^−1^ MY^−1^. In contrast, non-syntenic gene fixation rate was approximately 3.5 times higher for wheat 3B with a rate of 7.0 × 10^−3^ locus^−1^ MY^−1^ (Table 2). Although only partially sequenced, we found barley chromosome 3H to have a similar percentage of non-syntenic genes (31 %) and fixation rate (8.1 × 10^−3^ locus^−1^ MY^−1^) (Additional file 1: Table S3) as wheat 3B, suggesting that the high rate of interchromosomal duplication is a feature of the *Triticeae* lineage.

**Table 2:** Proportion of non-syntenic genes and their fixation rate for each species

Species	No. syntenic genes (%)	No. non-syntenic genes (%)	Divergence time (MYA)	non-syntenic gene fixation rate (locus^−1^ MY^−1^)
Ta3B	3,728 (72.7 %)	1,397(27.3 %)	39	7.0 × 10^−3^
Bd2	3,509 (92.2 %)	295 (7.8 %)	39	2.0 × 10^−3^
Os1	3,257 (90.9 %)	325 (9.1 %)	54	1.7 × 10^−3^
Sb3	3,472 (86.3 %)	551 (13.7 %)	60	2.3 × 10^−3^

Percentages are out of the total number of genes. Divergence times were taken from [12]

The contrasting levels of synteny between wheat and the other species studied here may reflect a major difference in evolutionary dynamics between small and large genomes. We performed the same synteny analysis with the regions of maize chromosomes 3 and 8 that are syntenic to wheat 3B. We found 8 % of the maize filtered genes to be non-syntenic (Additional file 1: Table S3), far from the 27 % in wheat. This gives further evidence that the high percentage of interchromosomal duplications is specific to the *Triticeae* lineage and not just a feature of large genomes.

We then compared the sequence of wheat chromosome 3B with the chromosome survey sequence assemblies of all chromosomes produced by the International Wheat Genome Sequencing Consortium [26], excluding 3A, 3B, and 3D. We found that 52 % of the Ta3B non-syntenic genes have at least one copy on a non-homeologous chromosome elsewhere in the wheat genome (BLASTN hit at ≥80 % identity and ≥50 % overlap). This is likely an underestimate due to the fact that the survey sequence dataset is not an exhaustive representation of the wheat gene set, and genes might be partially assembled or unannotated. Therefore, there is evidence that more than half of the non-syntenic genes originated from lineage-specific interchromosomal duplications. The remaining non-syntenic genes may be the result of translocations, missing sequence data, or duplications followed by loss of the ancestral locus. Thus, we conclude that interchromosomal duplications which occurred in the past 39 MY have contributed to the increased number of genes on wheat chromosome 3B compared to other grass species and that this pattern is likely a feature of the *Triticeae* lineage.

### Contrasted distribution of non-syntenic genes along the chromosome

**Fig. 3:**
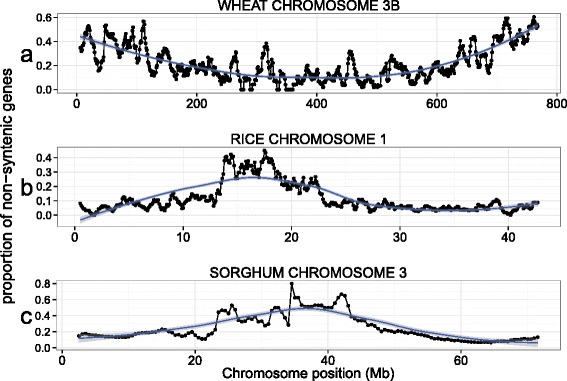
Chromosomal distributions of non-syntenic genes. Proportion of non-syntenic genes per total number of genes for (**a**) wheat chromosome 3B, (**b**) rice chromosome 1, and (**c**) sorghum chromosome 3. For wheat, the proportion of non-syntenic genes per total gene count was plotted in a sliding window of 10 Mb with a step of 1 Mb; in rice, 1 Mb sliding window with a step of 0.1 Mb, and for sorghum a 5 Mb sliding window with a step of 0.5 Mb. Pseudogenes were removed

The proportion of non-syntenic genes is positively correlated with the distance to the centromere (r = 0.6807) on wheat chromosome 3B. Interestingly, this pattern is opposite in the genomes of rice and sorghum in which a negative correlation is observed (r = −0.7279, r = −0.7797, respectively; Fig. 3). (*Brachypodium* was not used because the region syntenic to Ta3B only covers about half of the chromosome.) For barley 3H, we observed a similar pattern of non-syntenic gene distribution to that of wheat 3B (Additional file 1: Figure S5), suggesting that evolutionary forces governing such genome plasticity predate the divergence of the *Triticeae* lineage.

**Fig. 4:**
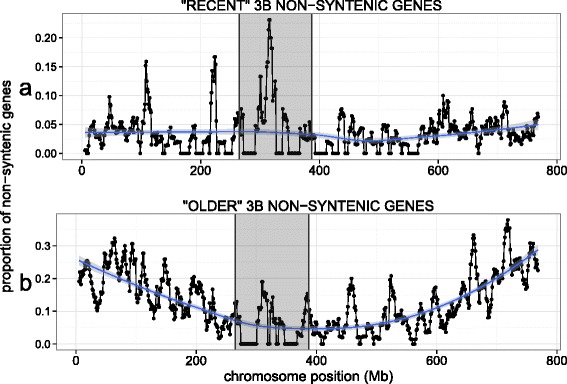
Comparison of the distributions of older versus more recent wheat non-syntenic genes along chromosome 3B. **a** “Recent” non-syntenic genes that moved after the divergence of the wheat progenitors (carried by 3B but without homolog on 3A, 3D, or 3H). **b** “Older” non-syntenic genes that have moved in the *Triticeae* lineage after divergence with *Brachypodium* and before the divergence of the wheat progenitors. These are 3B non-syntenic genes with a homolog on 3A, 3D, or 3H. The proportion of older and more recent non-syntenic genes out of the total number of genes was calculated within a 10 Mb sliding window with a step of 1 Mb. The centromeric/pericentromeric region is highlighted in the gray box

In this study, we define 3B non-syntenic genes as those genes originating from duplication or translocation events that occurred specifically in the wheat lineage after the divergence with *Brachypodium* approximately 39 MYA. In order to gain deeper insights into the more recent evolution history, we used the partially sequenced genomes of barley (common ancestor: approximately 11.6 MYA) and wheat homeologous chromosomes 3A and 3D from the chromosome survey sequence (common ancestor: approximately 6.5 MYA; Fig. 1). We distinguished a set of 3B-specific genes, representing 3B non-syntenic genes for which no ortholog was found on barley 3H and no homeolog on wheat chromosomes 3A or 3D. We compared these 3B-specific genes to the non-syntenic genes that were conserved with either 3A, 3D, or 3H. These two subclasses allowed us to distinguish ‘old’ and ‘recent’ non-syntenic genes. Old non-syntenic genes relocated before the divergence of the A/B/D progenitor species more than 6.5 MYA. We know from previous studies that intrachromosomal duplicates have a higher proportion at the distal regions of chromosome 3B [14], so we chose one random representative per duplicate family to avoid biasing the chromosomal distribution plot. In total, we found 161 recent (duplicated <6.5 MYA) and 738 old (duplicated between 6.5 and 39 MYA) non-syntenic gene representatives on the 3B pseudomolecule. Their distribution pattern revealed that the recent interchromosomal gene duplication and/or translocation events occurred on average at a similar rate along the chromosome (Fig. 4a). Interestingly, there is a spike in the centromeric region, suggesting that there may be a slight preferential insertion of non-syntenic genes into this region, as in rice and sorghum (Fig. 3). For older non-syntenic genes, the proportion increases towards the distal ends of the chromosome (Fig. 4b), corresponding to the regions where meiotic recombination mainly occurs [14].

### Non-syntenic genes are functional and may provide adaptive advantages

Non-syntenic genes appear to be significantly shorter in size than syntenic genes when considering gene length (introns + exons), coding sequence (CDS) length, and number of exons (Table 3). However, although the distribution of the syntenic gene CDS size has a higher upper tail (Additional file 1: Figure S6), the median CDS for syntenic and non-syntenic genes is nearly the same. Using deep transcriptome sequencing data from 15 different conditions (see Materials and Methods, [34]), we observed that 74 % of the non-syntenic genes are expressed in at least one condition (Table 3). Although this proportion is significantly lower than the 83 % of expressed syntenic genes, this indicates that the majority of non-syntenic genes are still functional and not only remnants of intense duplication activity. Interestingly, expressed non-syntenic genes are transcribed in fewer conditions on average (9 vs. 12 conditions) and at a lower intensity than syntenic genes (142 vs. 261 mean FPKM; Table 3 and Additional file 1: Figure S6). Forty-two percent of the genes expressed specifically in one condition are non-syntenic, whereas 84 % of those expressed constitutively are syntenic. Furthermore, non-syntenic genes have fewer numbers of alternative splice variants compared to syntenic genes (3.6 vs. 5.3) (Table 3). These results suggest that either non-syntenic genes have acquired a tissue-specific expression pattern through processes like subfunctionalization, or that genes expressed in a tissue-specific manner are more likely to be duplicated.

**Table 3:** Structural and functional features of the 3B syntenic and non-syntenic genes

	Syntenic	Non-syntenic	Significance
Mean genomic size	3,299 bp	3,008 bp	*P* value^a^ = 1.1e-4
Median genomic size	2,259 bp	1,907 bp	
Mean CDS size	1,252 bp	1,150 bp	*P* value^a^ = 8.3e-3
Median CDS size	1,070 bp	1,044 bp	
Mean number exons	4.8	3.6	*P* value^a^ = 3.2e-16
Median number exons	3	2	
Expressed	83 %	74 %	*P* value^b^ = 1.8e-13
Mean number conditions expressed^c^	12.0	9.2	*P* value^a^ <2.2e-16
Median number conditions expressed^c^	15	10	
Mean fpkm^c^	260.8	142.0	*P* value^a^ <2.2e-16
Median fpkm^c^	130.8	46.3	
Mean number alternative splice variants	5.3	3.6	*P* value^c^ <2.2e-16
Median number alternative splice variants	2	1	

Percentages are out of the total filtered gene count

^a^ Mann–Whitney-Wilcoxon test

^b^ Chi-squared test

^c^ Of those genes expressed

**Fig. 5:**
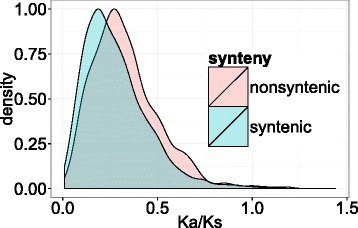
Frequency distributions of Ka/Ks rates of syntenic (blue) and non-syntenic genes (red). Coding sequences of 3,877 3B genes were aligned with their closest homolog in *Brachypodium*

We then investigated the selection pressure on non-syntenic versus syntenic genes by aligning their sequence with their closest homolog in *Brachypodium* to determine their synonymous and non-synonymous substitution rates since these species have diverged. We observed that non-syntenic genes have a significantly higher Ka/Ks compared to syntenic genes, indicating that non-syntenic genes are under a more relaxed selection pressure (Fig. 5).

Finally, we investigated the functions of non-syntenic genes in order to analyze the potential overrepresentation of specific categories. Gene ontology (GO) enrichment was analyzed for both syntenic genes and non-syntenic genes, and several GO terms were found to be significantly overrepresented in both types of genes. Syntenic genes were enriched in biological processes that are essential, metabolic functions, particularly: regulation of primary metabolic process, regulation of gene expression, regulation of cellular biosynthetic process, nucleobase-containing compound metabolic process, gene expression, among others (Table 4). In contrast, non-syntenic genes were enriched in far fewer categories: programmed cell death and macromolecule modification (Table 4) thereby suggesting that non-syntenic genes may provide some adaptive advantages to biotic or abiotic factors.

**Table 4:** GO enrichment analysis of Biological Process terms for syntenic genes and non-syntenic genes

**Syntenic genes**						
GO ID	Term	Annotated	Significant	Expected	*P* value	Adjusted *P* value
GO:0019219	Regulation of nucleobase-containing compound metabolic process	372	229	176.14	4.3e-09	8.2e-07
GO:0019222	Regulation of metabolic process	437	263	206.92	6.2e-09	9.9e-07
GO:0031323	Regulation of cellular metabolic process	382	232	180.88	1.9e-08	2.7e-06
GO:0060255	Regulation of macromolecule metabolic process	404	243	191.3	2.9e-08	3.67e-06
GO:0010468	Regulation of gene expression	378	228	178.98	6.0e-08	5.97e-06
GO:0009889	Regulation of biosynthetic process	373	225	176.62	7.4e-08	5.97e-06
GO:0010556	Regulation of macromolecule biosynthetic process	373	225	176.62	7.4e-08	5.97e-06
GO:0031326	Regulation of cellular biosynthetic process	373	225	176.62	7.4e-08	5.97e-06
GO:0006139	Nucleobase-containing compound metabolic process	695	391	329.09	9.7e-08	6.8e-06
GO:0010467	Gene expression	679	378	321.51	8.5e-07	5.0e-05
GO:0044248	Cellular catabolic process	163	100	77.18	0.00017	0.0090
GO:1901576	Organic substance catabolic process	178	106	84.28	0.00054	2.1639e-02
GO:0044249	Cellular biosynthetic process	742	393	351.34	0.00033	1.5428e-02
GO:0016485	Protein processing	100	64	47.35	0.00050	2.0778e-02
GO:0071704	Organic substance metabolic process	2000	998	947.01	0.00029	1.4790e-02
GO:0009058	Biosynthetic process	750	396	355.13	0.00044	1.9747e-02
GO:0015672	Monovalent inorganic cation transport	41	30	19.41	0.00067	2.3120e-02
GO:0009057	Macromolecule catabolic process	146	87	69.13	0.00163	4.6894e-02
GO:0006812	Cation transport	67	44	31.72	0.00175	4.9087e-02
GO:0019941	Modification-dependent protein catabolic process	97	62	45.93	0.00064	2.3120e-02
GO:0070647	Protein modification by small protein conjugation or removal	99	62	46.88	0.00139	4.1042e-02
**Non-syntenic genes**						
GO ID	Term	Annotated	Significant	Expected	*P* value	Adjusted *P* value
GO:0012501	Programmed cell death	172	53	30.12	8.5e-06	0.00374
GO:0043412	Macromolecule modification	663	150	116.1	0.00011	0.030855

## Discussion

Our comparative analyses of a stringently filtered core set of genes reveal that wheat chromosome 3B carries a significantly higher number of genes than was previously expected based on the number of genes in the model grass species rice, *Brachypodium*, and sorghum. We are confident that the 5,125 genes filtered from the wheat chromosome 3B sequence correspond to real genes rather than transposable elements, mispredicted genes, or pseudogenes and therefore, that the observation is robust. This is because of four reasons: (1) TEs were previously annotated on 3B and removed from the wheat gene set [14]; (2) all of these genes show similarity to at least one other gene in rice, *Brachypodium*, or sorghum; (3) 95 % of the genes show similarity to *A. thaliana* (Additional file 1: Table S1), in accordance with the expected 11 % of genes to be monocot-specific [35]; and (4) pseudogenes (genes with nonsense mutations or large deletions) were removed from the dataset. This unusually high amount of genes indicates major single gene duplication activity during the evolution of the wheat genome over the past 39 MY.

The higher number of genes in the wheat 3B core gene set compared to other grass species most likely holds true at the whole genome level, as confirmed by the prediction of 44,523 high confidence genes in the B subgenome chromosome survey sequence assemblies [26]. This is the highest number of protein-coding genes observed for a diploid grass genome (Brachypodium: 26,552 genes; sorghum: 34,496; rice: 39,045; maize: 39,389). These small scale interchromosomal duplications, together with an increased proportion of intrachromosomal duplications [14] and two rounds of polyploidization have led to a highly redundant genome, providing a rich arsenal of raw genetic material and potential means of adaptation.

### The wheat genome has undergone more interchromosomal duplications than related grasses

The definitions of synteny and collinearity have become blurred in recent years. In this paper, we make the distinction as follows: two genes in different species are syntenic if they are conserved on their corresponding orthologous chromosome, and collinear if they are on their corresponding chromosome and with a preserved gene order. Thus, collinearity is a more stringent form of synteny, and detects gene duplication and movement on the same chromosome. This is consistent with the original definition of synteny [36, 37]. Because we were only concerned with studying the extent of interchromosomal duplication (genes which have moved to a different chromosome in their specific lineage), we only considered synteny and conservation of overall orthologous chromosomal location.

We quantified the proportion of non-syntenic genes (27 %), and determined that most of these originated from interchromosomal duplications. Previous estimates using partial sequence information were in the range of 41–48 % for wheat chromosome 3B [18, 23]. Our estimation of 27 % is likely to be an underestimate due to our stringent filtration process in order to focus only on a high confidence core gene set. Even when considering only non-syntenic genes defined by our stringent criteria, wheat has more than double the proportion of non-syntenic genes (about 10 % vs. 27 %), and a three-fold duplication/retention rate (2 × 10^−3^ vs. 7.0 × 10^−3^) than the three other grasses analyzed here. In addition, the rate in sorghum is potentially overestimated since it is the outgroup of the four species used in this study. Thus we cannot distinguish between genes that relocated in the sorghum lineage and genes which were lost in the common ancestor of rice/*Brachypodium*/wheat.

This ‘accelerated evolution’ in the wheat lineage was previously observed in the D genome progenitor, *Ae. tauschii*, through the analysis of large scale genomic rearrangements [24]. As similar results were found for barley chromosome 3H, there is evidence that the increased rate of gene duplication is a feature of the *Triticeae* lineage. It will be interesting to perform similar analyses in rye to confirm when single gene duplications have started to increase the gene number in the *Triticeae* lineage. This increased rate of duplication supports the previously described notion of accelerated evolution in the wheat genome based on the increased number of alternative splicing events, non-synonymous substitution rates, and gene exon rearrangements compared to other grass lineages [25].

### Potential mechanisms of interchromosomal gene movement

Single gene duplication can originate from different mechanisms. Retroposition, where genes are reverse-transcribed and reinserted back into the genome, has been shown to play a role in gene creation in plants [38, 39]. Alternatively, there has been a growing amount of evidence that long-distance gene movement may be the result of ectopic recombination during the process of double strand break (DSB) repair [40]. In grasses, it has been suggested that gene movement is most likely due to synthesis-dependent strand annealing upon DSB repair [41]. In addition, genes can be also be created and rearranged in genomes by TE-mediated transposon capture or exon shuffling [42]. Indeed, several studies have demonstrated that genes can be captured in TEs and moved throughout plant genomes [43–46]. This phenomenon has been demonstrated on wheat chromosome 3B, where CACTA transposons were shown to have captured and moved 140 of the non-syntenic genes [32]. However, this only explains a small percentage of the 5,125 non-syntenic genes. Upon duplication, one copy of the gene may turn into a pseudogene [47], or the duplicated gene may be retained, possibly undergoing sub- or neo-functionalization of one or both copies [48]. More studies are needed to precisely investigate which one of the potential mechanisms of interchromosomal gene movement led to the increased proportions we observe on wheat 3B.

### Chromosomal distribution patterns of non-syntenic genes

Our finding of an increased proportion of retained non-syntenic genes in the distal regions of wheat chromosome 3B (Fig. 4) and barley chromosome 3H confirms previous studies [22, 23, 49]. When looking at the most recently duplicated genes (3B-specific non-syntenic genes), we found no such gradient, suggesting that non-syntenic genes are not preferentially inserted at the distal regions of the chromosome but that their elimination is biased. The spike in the proportion of 3B-specific non-syntenic genes observed in the centromeric region of chromosome 3B is similar to what is found in rice and sorghum chromosomes and support previous observations that synteny correlates with recombination [50]. As discussed in [51], a potential explanation for the difference in the distribution of syntenic vs. non-syntenic genes is that for species with a lower degree of synteny at their centromeric region, there may be more loss of single-gene duplications in euchromatin regions compared to heterochromatic regions due to the lack of recombination near the centromere. This in turn may preserve duplicated genes in the pericentromeric regions [50]. In addition, non-syntenic genes may not be counter-selected in the pericentromeric regions because of the low gene density in these areas (insertions being less deleterious than in areas of high gene density) [51].

Interestingly, this pattern contrasts with the distribution of the ‘older’ Ta3B non-syntenic genes (>6.5 MY), that increase in proportion towards the ends of the chromosome. So, which evolutionary forces shaped this pattern? Recombination as well as gene density increases with relative distance from the centromere, and intrachromosomally duplicated genes are found more frequently at the distal regions of the chromosome [14, 28]. The higher recombination rate at the distal regions could in turn promote rapid evolution [49]. On wheat 3B, DNA transposons represent 18 % of the sequence and tend to be located near the distal regions of the chromosome [32]. These small, repetitive TEs could serve as homologous sequences for unequal recombination, which could in turn can generate duplicated genes [2]. Previous studies found that, in *Triticeae*, the level of gene sequence polymorphism increases towards the distal regions [52]. This is also supported in Drosophila where beneficial mutations are fixed more efficiently in the high-recombination regions [53]. Thus, in wheat, the probability for a new gene to be retained seems to be higher in distal regions, and this may be due to an interplay of a number of factors, including gene density, TE content, and recombination.

### Fate of non-syntenic genes

A previous study using draft sequences of individual chromosomes from wheat group 1 [54] observed a high amount of non-syntenic genes and concluded that many of these are pseudogenes. Here, we show that on chromosome 3B, even upon removing pseudogenes in the filtered set, the majority of the non-syntenic genes are expressed (74 %), indicating that most of these non-syntenic genes are functional. We also observed that older, syntenic, more conserved genes are more constitutively expressed and at a higher level than younger, non-syntenic genes. This supports other findings showing that recently evolved genes have a narrower expression range and level [55] than older and more conserved genes. Genes expressed in a broad range of tissues may evolve slower because the sequence divergence is restricted due the pleiotropic effects of many proteins interacting with each other [56].

Previous studies have shown that alternative splicing is reduced in duplicated genes shortly after they have been duplicated [57, 58]. The authors postulate that since duplicated genes are known to diverge in expression pattern, the reduction of alternative splicing capabilities, and thus, protein functional diversity, in young duplicates is compensated for by subfunctionalization. We also found that non-syntenic genes have fewer isoforms than syntenic genes, which supports this hypothesis. Additionally, duplicated genes in particular are known to evolve much faster than singletons due to sub- or neo-functionalization [59] and may provide adaptive advantages [60].

Although using GO for gene enrichment analysis has some inherent limitations and common drawbacks, it can also be a powerful tool for functional profiling [61]. We found that 3B syntenic genes tend to be enriched in biological processes that are essential, metabolic functions, whereas non-syntenic genes were enriched in processes that could provide some sort of adaptive advantages against biotic or abiotic factors, such as disease resistance. In this study we only investigated syntenic and non-syntenic genes on chromosome 3B, thus the enrichment was normalized by using only 3B as a reference, rather than the entire genome. The question still remains if non-syntenic and syntenic genes will be enriched for the same functions on the remaining chromosomes in the genome. Furthermore, the GO annotations for 3B were inferred computationally, so further experimental evidence will be necessary to confirm these functions.

Nevertheless, the most significantly enriched GO term for non-syntenic genes was programmed cell death (Table 4), which is a known plant defense strategy by which specific cells are destroyed, keeping neighboring cells intact. Programmed cell death can be induced by drought, salt, high temperatures, and other abiotic stresses [62], and is a common mechanism of pathogen resistance in plants [63]. Interestingly, wheat disease resistance genes have been shown to be located near the distal regions of the chromosomes [64]. Additionally, there is evidence that many cereal disease resistance genes are likely non-syntenic [65]. A recent study of mammalian genomes found that the proportion of genes having undergone small scale duplication was correlated with habitat variability, suggesting that species in variable habitats maintain small scale duplications as a way to adapt to their environment [66].

## Conclusions

This in-depth analysis of interchromosomal duplications on the reference sequence of chromosome 3B enabled us to study non-syntenic genes between wheat, rice, *Brachypodium*, and sorghum at the highest resolution to date. The 3B pseudomolecule provides a valuable resource for studies of duplicated genes-- the BAC-by-BAC sequencing and assembly technique allows for fewer collapsed duplicated genes (assemblies with several paralogs merged into chimeric sequences). Thus we have obtained a more accurate picture of interchromosomal gene duplication in the wheat genome.

We performed a stringent analysis with many filtering steps in order to avoid spurious gene annotations. For the first time, we show that even without pseudogenes, the wheat genome has a higher proportion of non-syntenic genes compared to rice, *Brachypodium*, and sorghum. These non-syntenic genes have nearly the same coding sequence length as the syntenic genes, and the majority are expressed, showing that these are not ‘dead on arrival’, but functional (albeit with a more relaxed selection pressure). We show that non-syntenic genes are not preferentially inserted at the distal regions, but rather are selected for there.

This study provides another piece evidence for accelerated evolution in the *Triticeae* lineage. This accelerated evolution is in the form of massive interchromosomal duplications, and resulted in a higher number of genes than other grass species and an increased potential for gene adaption. Although polyploidization is one route towards adaptation, single gene duplications that have been occurring before and after the hybridization events have also greatly contributed to inflating the wheat gene repertoire. The structural and functional redundancy provided by the high duplication activity in the wheat genome has likely provided a selective advantage to wheat for adapting to a large range of environments, making it one of the most successful crops.

## Methods

### Species used and gene filtration

We downloaded the *Brachypodium* MIPS v1.2 (available at [67]), rice MSU v7 (available at ftp.plantbiology.msu.edu/), and sorghum v8.0 (available at ftp.jgi-psf.org) protein annotations for sequence comparisons. We delineated the regions of synteny between species by visualizing it with Ensembl Plants release 24 Synteny viewer [30]. The entire chromosomes of wheat 3B, rice 1, sorghum 3, and the distal regions of *Brachypodium* chromosome 2 are syntenic (Additional file 1: Figures S1-S4). For *Brachypodium* chromosome 2, we determined the borders of the syntenic distal regions: from the telomere of short arm to position 12,348,272 bp and from position 40,348,989 to the telomere of the long arm. In addition, we used the recently released *Hordeum vulgare* (barley) draft genome sequence [68] to compare to wheat as another representative of the *Triticeae* lineage. However, we did not use this draft for classifying Ta3B syntenic vs. non-syntenic genes because of the partial representation of the genome, with 2478 genes (less than half expected based on comparison with the other grasses) anchored onto chromosome 3H (syntenic with Ta3B).

The construction of a pseudomolecule for the wheat chromosome 3B was previously described in [14] and the chromosome survey sequence assemblies of each of the 20 other chromosomes were described in [26]. Genes from wheat 3B, rice 1, *Brachypodium* 2, sorghum 3, and barley 3H were subject to two rounds of filtration: we removed alternative splice variants (taking one representative model for each locus), all genes annotated as related to transposons, and, finally, all genes from the dataset that did not have a significant BLASTP hit (e-value ≤ 1e-5 with ≥35 % amino acid identity and ≥35 % sequence overlap).

Pseudogenes were removed from the dataset as follows: genes were defined as pseudogenes if their model contained internal stop codons, frame shift mutations, or deletions (leaving less than 70 % of the length of a complete homolog) within the CDS.

### Classification of syntenic vs. non-syntenic genes

In order to identify syntenic and non-syntenic genes, amino acid sequences of the entire gene set of all species considered were compared using all-by-all BLAST. The best BLAST hit (e-value cutoff 1e-5) in each species was identified for each gene. We used best BLAST hit rather than reciprocal best hits to infer orthologs because in highly duplicated genomes such as wheat, methods based on reciprocal best hits have a high rate of false negatives and will miss many of the true orthologs [69]. If a gene had at least one best hit in another species on a syntenic counterpart, it was classified as syntenic. If the gene had all best BLAST hits carried by non-syntenic chromosomes, it was classified as non-syntenic. The non-syntenic gene fixation rate for each species was calculated by the number of non-syntenic genes/total number of genes in the genome/millions of years since the divergence from the last common ancestor. Non-syntenic genes were determined to have originated by duplication events if a nucleotide BLAST hit (at least 80 % identity and 50 % overlap) was found on a non-homeologous chromosome (not 3A, 3B, or 3D) of the chromosome survey sequence.

### Distributions along the chromosomes

For wheat, the chromosome distribution of non-syntenic genes was performed by calculating the proportion of non-syntenic genes/total number of genes within a window of 10 Mb sliding at 1 Mb along the chromosome. For rice, this was a window of 1 Mb sliding at 0.1 Mb, and for sorghum 5 Mb window, sliding at 0.5 Mb. Centromere locations were estimated based on [10, 12, 14, 70]. R was used for calculation of the Pearson correlation coefficients. The loess function in ggplot2 was used to draw the curve.

### Calculation of non-synonymous (Ka) and synonymous (Ks) substitution rates

We used the coding sequences of non-syntenic genes and their best BLASTP hit in *Brachypodium distachyon* to make an alignment with TranslatorX [71] for calculating Ka and Ks. Rates were calculated by the Nei and Gojobori method using codeml (part of the PAML package; [72]). This resulted in Ka and Ks rates for 3,962 pairs after removing those with a frameshift or stop codon mutation. To calculate the Ka/Ks ratio, we removed the samples that had Ks of 0, leaving 3,866 pairs.

### Gene expression analysis

RNA extraction, library construction and sequencing was described in [14]. Briefly, total RNAs were extracted in duplicates from the wheat cv. Chinese Spring using five organs (root, leaf, stem, spike, and grain) at three developmental stages each. IlluminaTruSeq^TM^ RNA sample preparation Kit (Illumina, USA) was used to create non-oriented RNA-seq libraries (4 μg of total RNA used with a library insert fragmentation time of 12 min). Libraries were sequenced on an Illumina HiSeq2000 with 2 × 100-bp paired-end reads. Read quality was verified using FastQC v0.10.0 [73]. Illumina reads were mapped to chromosome 3B using Tophat2 v2.0.8 [74] and bowtie2 [75] with the default parameters except: 0 mismatch, 0 splice-mismatch. PCR duplicates were removed with Samtools [76] with the rmdup option and an annotation-guided read alignment was performed with Cufflinks v2.1.1 [77] to reassemble and quantify transcripts in units of fragments per kb of exon per million mapped reads (FPKM). Expressed regions were considered as those with an FPKM higher than zero. Hierarchical clustering was performed using the Hierarchical Clustering Explorer 3.5 software [78] with the complete linkage method and the Pearson correlation coefficient. FPKM values were log2 transformed (log2(FPKM + 1)). The minimal similarity to establish the clusters was set to 0.641 which is the Pearson correlation significant at the *P* value threshold of 0.01.

### Gene Ontology term enrichment analysis

Gene ontology (GO) term enrichment was described in [14]. Briefly, similarity searches using BLASTP (e-value < 1e-05) were performed for each amino acid sequence, against the PLAZA 2.5 protein database [79]. Consensus functional information was assigned to the 3B gene products based on homolog GO or InterPro information. Only the five best homologs with more than 50 % coverage were used for the analysis. The topGO R package was used for enrichment calculations [80]. The full set of 3B gene products was used as the reference comparison set against non-syntenic and syntenic gene sets. *P* values were calculated using Fisher’s exact test and they were corrected for multiple testing with FDR method using the R module called ‘p-adjust’. Finally, the redundancy from the list of enriched GO terms was removed using the program GO Trimming [81] using default parameters.

## Additional file

Additional file 1: Table S1. Comparison of wheat filtered gene set to *Arabidopsis* gene set. **Table S2.** Genes reclassified from non-syntenic to syntenic genes by BLAST against genomic DNA of orthologous chromosomes. **Table S3** Synteny analysis of barley 3H and maize chromosomes 3 and 8 (regions syntenic to wheat 3B). **Figures S1-S4.** Rice, *Brachypodium*, sorghum, and wheat syntenic chromosomes, respectively. **Figure S5.** Proportion of non-syntenic genes per total genes for barley chromosome 3H. (PDF 759 kb)

## Abbreviations

ASV: alternative splice variant
BAC: bacteria artificial chromosome
Bd2: Brachypodium chromosome 2
CDS: coding sequence
DSB: double-strand break
EST: expressed sequence tag
FPKM: Fragments Per Kilobase of transcript per Million mapped reads
GO: gene ontology
Ka: non-synonymous substitution rate
Ks: synonymous substitution rate
MYA: million years ago
Os1: rice chromosome 1
Sb3: sorghum chromosome 3
Ta3B: wheat chromosome 3B
TE: transposable element
