# Supplementary material for: Small-scale gene duplications played a major role in the recent evolution of wheat chromosome 3B
Source: Genome Biol. 2015 Sep 9;16(1):188. doi: 10.1186/s13059-015-0754-6 (PMC4563886; doi:10.1186/s13059-015-0754-6)
Supplement: Additional file 1: Table S1. — Comparison of wheat filtered gene set to Arabidopsis gene set. Table S2. Genes reclassified from non-syntenic to syntenic genes by BLAST against genomic DNA of orthologous chromosomes. Table S3 Synteny analysis of barley 3H and maize chromosomes 3 and 8 (regions syntenic to wheat 3B). Figures S1-S4. Rice, Brachypodium, sorghum, and wheat syntenic chromosomes, respectively. Figure S5. Proportion of non-syntenic genes per total genes for barley chromosome 3H. (PDF 759 kb) [file 13059_2015_754_MOESM1_ESM.pdf]

Table S1. Comparison of wheat filtered gene set to *Arabidopsis* gene set.

	No BLAST hit $1e^{-5}$ e-value	No BLAST hit $1e^{-5}$ e-value, >35% overlap, >35% identity	No BLAST hit $1e^{-5}$ e-value , >50% overlap, >35% identity
All	112 (2.2%)	147 (2.9%)	252 (4.9%)
Syntenic	73	108	189
Nonsyntenic	39	39	63

BLASTP similarity search results of the wheat filtered gene set (5125 genes) against the *Arabidopsis thaliana* genome (TAIR version 10). The columns show increasing levels of stringency for identification of a homolog in the Arabidopsis genome. Note that even at the highest stringency (e-value=1e-5 & gene overlap=50% coverage & percent identity=35%), only 5% of the filtered genes do not have a match in Arabidopsis.

Table S2. Genes reclassified from nonsyntenic to syntenic genes by BLAST against genomic DNA of orthologous chromosomes. The percentage of false positives are the number of reclassified genes/ total number of genes for Ta3B, Os1, Bd2, or Sb3.

**Species**	**Number of genes reclassified**	**% nonsyntenic gene false positives**
Wheat	140	2.7%
Rice	70	2.0%
Brachypodium	92	2.4%
Sorghum	75	1.9%

Table S3. Synteny analysis of barley 3H and maize chromosomes 3 and 8 (regions syntenic to wheat 3B)

Species	No. SG (%)	No. NSG (%)	Divergence with most recent common ancestor (MYA)	NSG fixation rate (locus-1 MY-1)
Hordeum vulgare 3H	1699 (69%)	779 (31%)	39	8.1 x 10-3
Zea mays chr 3 & 8	3476 (92%)	295 (8%)	60	1.3 x 10-3

Estimated percentage of syntenic genes (SG) and nonsyntenic genes (NSG) on barley 3H, and maize chromosome 3 (31260777-69380680; 143725574-228989858) and chromosome 8 (17505-29882922; 33876556-47314747; 58054834-61802187; 135820185-175288181). Coordinates of syntenic regions were found using Ensembl Compara. Nonsyntenic gene fixation rates are also shown.

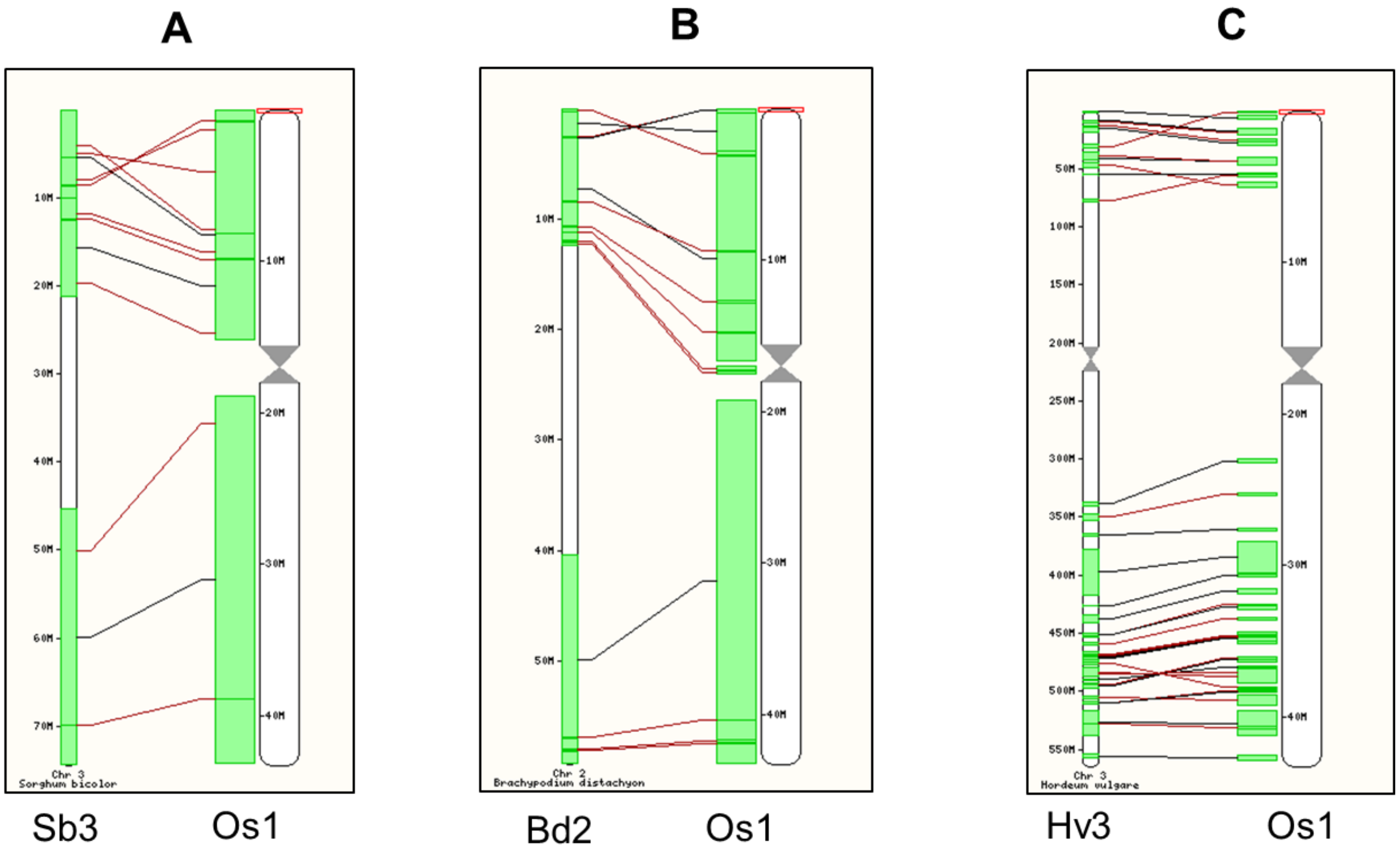

Figure S1. Rice syntenic chromosomes. A) Rice chromosome 1 (*Oryza sativa Japonica*; Os1) and syntenic regions detected from Sorghum (Sb). B) Os1 and syntenic regions detected from *B. distachyon* (Bd). C) Os1 and syntenic regions detected from barley (Hv). Chromosome numbers are indicated after the species abbreviation. Synteny images were downloaded from Ensembl Plants release 24 (http://plants.ensembl.org/index.html). Note that all syntenic chromosomes to the chromosome of interest are displayed in Ensembl are shown here.

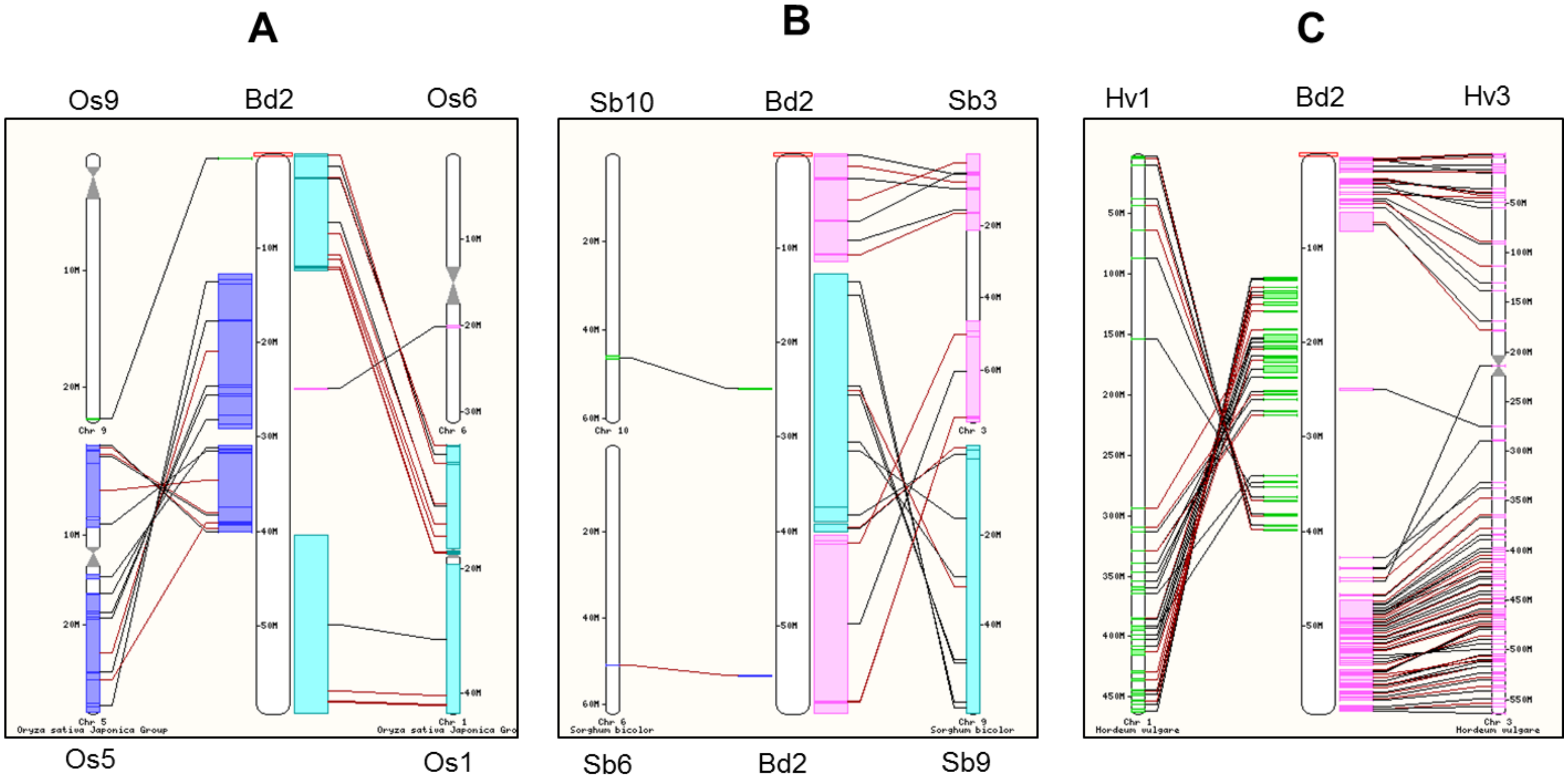

Figure S2. ***B. distachyon*** **syntenic chromosomes.** A) *B. distachyon* chromosome 2 (Bd2) and syntenic regions detected from rice (Os). B) Bd2 and syntenic regions detected from sorghum (Sb). C) Bd2 and syntenic regions detected from barley (Hv). Chromosome numbers are indicated after the species abbreviation. Synteny images were downloaded from Ensembl Plants release 24 (http://plants.ensembl.org/index.html). Note that all syntenic chromosomes to the chromosome of interest are displayed in Ensembl are shown here.

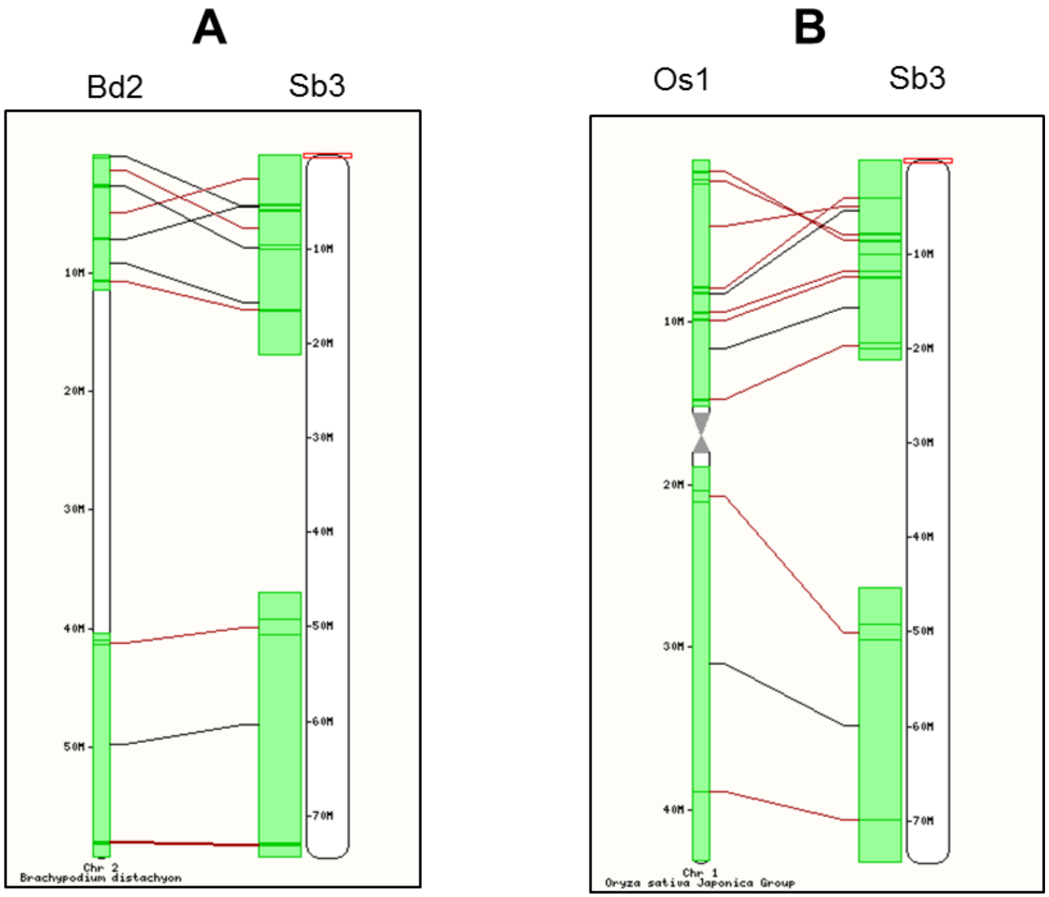

Figure S3. Sorghum syntenic chromosomes. A) *Sorghum bicolor* chromosome 3 (Sb3) and syntenic regions detected from *B. distachyon* (Bd). B) Sb3 and syntenic regions detected from rice (Os). Chromosome numbers are indicated after the species abbreviation. Synteny images were downloaded from Ensembl Plants release 24 (http://plants.ensembl.org/index.html). Note that all syntenic chromosomes to the chromosome of interest are displayed in Ensembl are shown here.

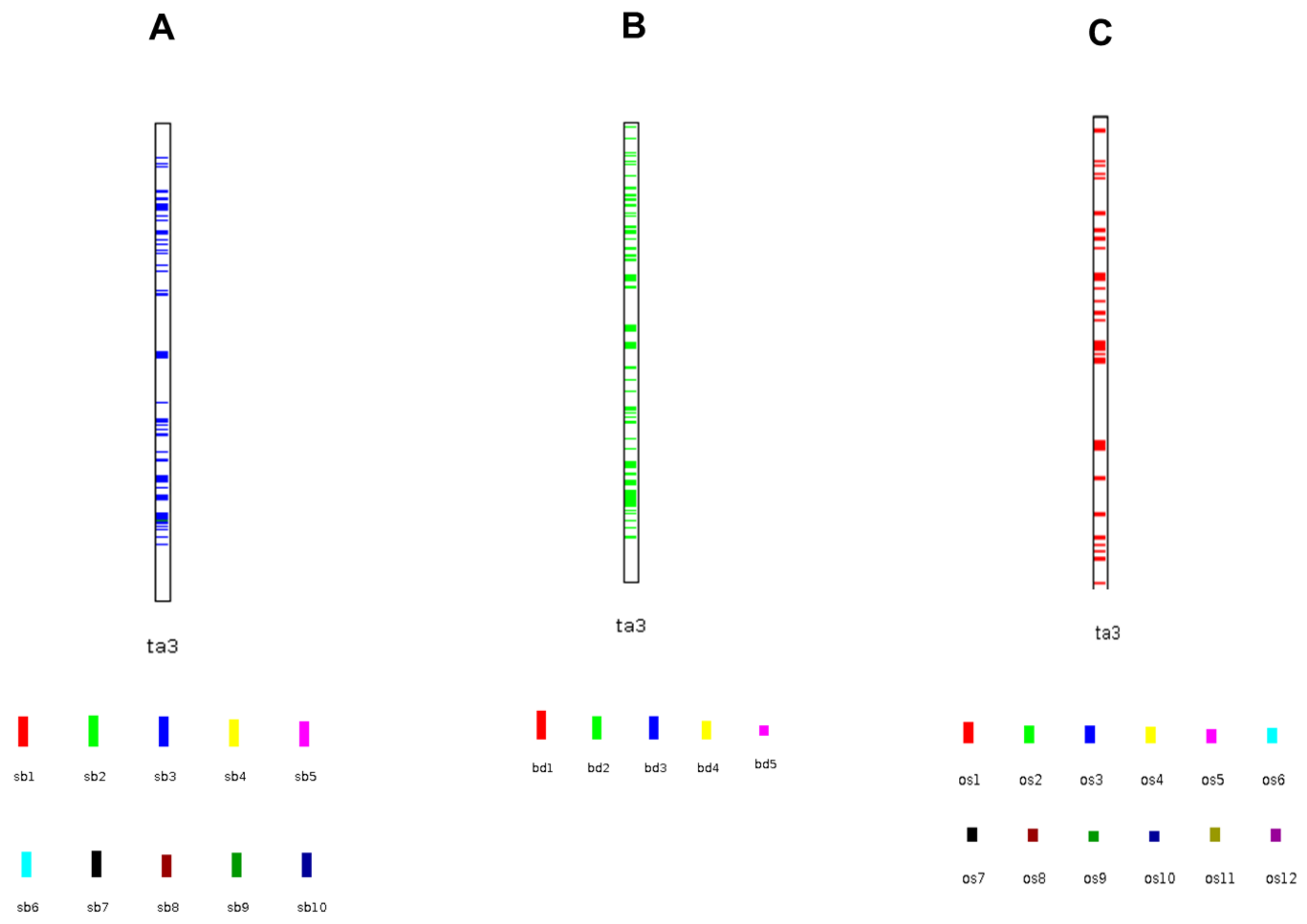
A
B
C
ta3
ta3
ta3
sb1
sb2
sb3
sb4
sb5
sb6
sb7
sb8
sb9
sb10
bd1
bd2
bd3
bd4
bd5
os1
os2
os3
os4
os5
os6
os7
os8
os9
os10
os11
os12

Figure S4. Wheat syntenic chromosomes. A) *Triticum aestivum* chromosome 3B (ta3) and syntenic regions detected from sorghum (sb). B) Ta3B and syntenic regions detected from *B. distachyon* (bd). C) Ta3B and syntenic regions detected from rice (os). Synteny calculation and visualization was done with the bar plotter tool in MCScanX.

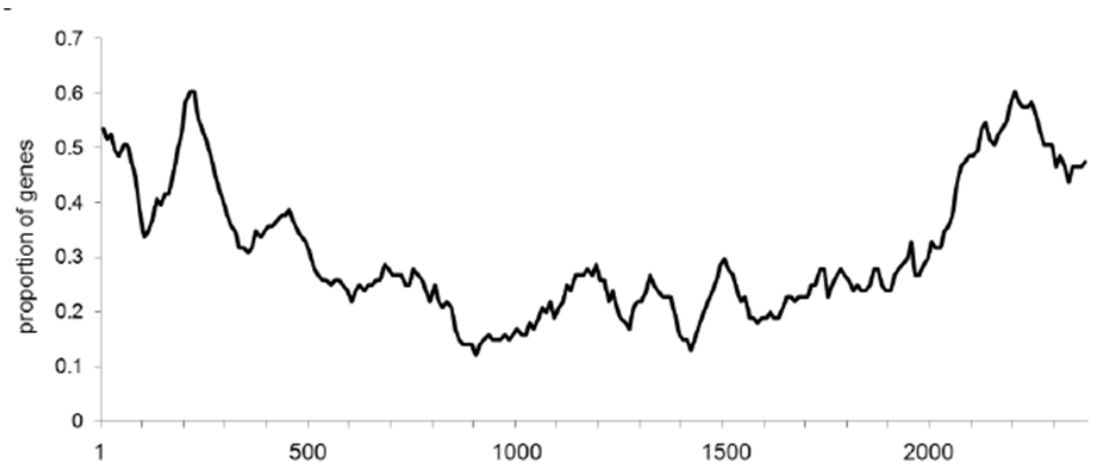

Figure S5. Proportion of NSGs per total genes for barley chromosome 3H. The proportion of NSGs per total gene count in a window of 100 genes (sliding at 10 genes) are plotted.

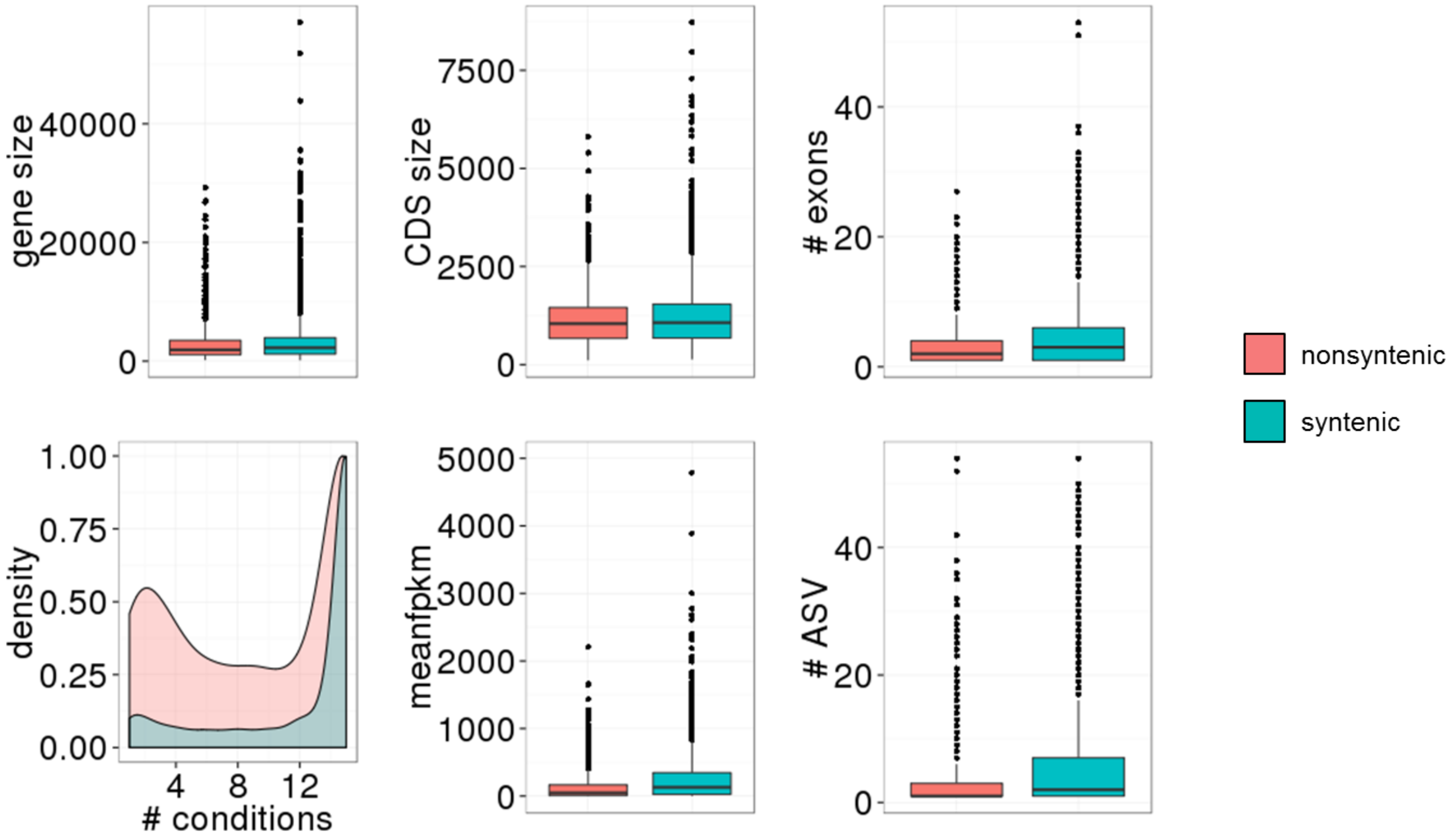

gene size
CDS size
exons
nonsyntenic
syntenic
density
conditions
meanfpkm
ASV

Figure S6. Comparisons of gene features between nonsyntenic (NSG) and syntenic (SG) genes. Box plots are provided for genomic sequence size, CDS size, number of exons, mean fpkm expression level, and number of alternative splice variants. The density plot shows the number of conditions expressed for nonsyntenic (pink) vs. syntenic (blue) genes. N=5125. The upper quartile, median, and lower quartile are represented by the upper, middle, and lower lines, respectively.
